# Cardiovascular Involvement of a CD138-Negative Anaplastic Myeloma: A Diagnostic Dilemma

**DOI:** 10.3390/hematolrep15010007

**Published:** 2023-01-18

**Authors:** Jui Choudhuri, Victor Janmey, Juan Ding, Denise Dailey, Yang Shi, Yanhua Wang

**Affiliations:** 1 Department of Pathology, Montefiore Medical Center, 111 E 210th Street, New York, NY 10467, USA; 2 Department of Radiology, Montefiore Medical Center, 111 E 210th Street, New York, NY 10467, USA

**Keywords:** myeloma, anaplastic, plasmacytoid, cardiac

## Abstract

Anaplastic myeloma (AM) is an extremely rare and aggressive histological variant of myeloma. It is characterized by extramedullary presentation in the young and has a poor prognosis. It can be a diagnostic challenge when myeloma is not suspected and even more when the immunophenotype is unexpected. We present a rare presentation of anaplastic myeloma with cardiovascular involvement. Though the patient did not have the typical clinical features of myeloma, except lytic lesion in the femur, the cardiac biopsy showed sheets of anaplastic cells, and some with multinucleation. There were also some areas with a more plasmacytoid appearance. The initial immunohistochemical panel was negative for CD3, CD20, CD138, AE1/3, and kappa. It was positive for lambda. This led to an extended panel which showed positivity for CD79a and MUM1 and negative for LMP-1, HHV-8, CD43, CD117, CD56, and CD30. Even the flow cytometry on the bone marrow showed a small population of atypical cells positive for CD38 and negative for CD138 with lambda restriction. This is an unusual case of anaplastic myeloma with cardiovascular involvement and CD138 negativity. This case highlights the need to add a panel of plasma cell markers when myeloma is suspected, and it is pertinent to read flow cytometry with caution to avoid missing atypical plasma cells which maybe CD38+/CD138−.

## 1. Introduction

Anaplastic myeloma is a rare, aggressive form of myeloma characterized by poorly differentiated plasma cells. The first case was reported in 1983 by Foucar et al. and it has since been reported sporadically in case reports [1,2,3,4,5]. This morphological variant of myeloma is characteristically more frequent in young patients with extramedullary disease presentation, IgA isotype, and refractoriness to therapy. Anaplastic morphology may be present at initial diagnosis or as a feature of progression from plasma cell myeloma, these include large, pleomorphic, multinucleated cells and these may resemble dysplastic megakaryocytes in a bone marrow biopsy. These cells may have moderate to abundant basophilic cytoplasm, prominent nucleoli, and intranuclear basophilic inclusions [2,3,6]. Anaplastic morphology encountered in newly diagnosed cases poses a major diagnostic challenge and even more in cases with unusual phenotypes. We present an unusual case of anaplastic myeloma with a unique presentation clinically and immunophenotypically.

## 2. Case Presentation 

A 69-year-old male presented to the oncology clinic with referral for a right maxillary sinus mass. He presented with severe right sided jaw and leg pain for five weeks. His past medical history was significant for diabetes mellitus, hypertension, chronic kidney disease (grade III), and coronary artery disease. He had undergone multiple angioplasties in the past. The physical examination was unremarkable. At presentation, his white blood cell count was 11.3 k/uL (Reference 4.8–10.8 k/uL), with 68% neutrophils, 21% lymphocytes, 7% monocytes and 4% eosinophils. His hemoglobin was 11.2 g/dL (Reference 14–17.4 g/dL) and platelet count was 263 k/uL (Reference 150–400 k/uL). His biochemical parameters were unremarkable except elevated creatinine of 1.44 mg/dL (Reference < 1.30 mg/dL). No monoclonal protein was identified in initial serum protein electrophoresis and the patient had hypogammaglobulinemia. Serum IgG, IgA, and IgM levels were 630 (Reference 700–1600 mg/dL), 171.0 (Reference 60.0–400.0 mg/dL), and 93.0 mg/dL (Reference 50.0–300.0 mg/dL), respectively. Serum free light chain showed an increased free lambda light chain of 71.8 mg/L (Reference 5.7–26.3 mg/L) and normal kappa light chain of 20.3 mg /L (Reference 3.3–19.4 mg/L), with kappa lambda light chain ratio of 0.28 (20.3/71.8). Beta-2 microglobulin was elevated to 4.0 ng/mL (Reference 1.0–3.0 ng/mL). The patient tested negative for human immunodeficiency virus (HIV) infection. 

**Figure 1:**
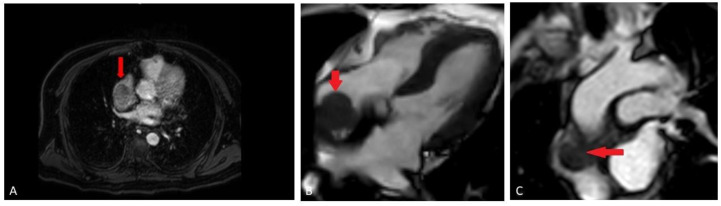
(**A**) Cardiac MRI early post-contrast mDixon sequence (fat suppressed gradient echo sequence), demonstrates enhancing component of the mass in the distal left Superior Vena Cava (SVC); (**B**) cardiac MRI pre-contrast balanced turbo field echo (BTFE) sequence, demonstrating adherent mass (red arrow) along the right atrial free wall; (**C**) cardiac MRI early post-contrast perfusion sequence (T1 weighted, gradient-echo), demonstrating relatively homogeneous enhancement of the mass (red arrow).

**Figure 2:**
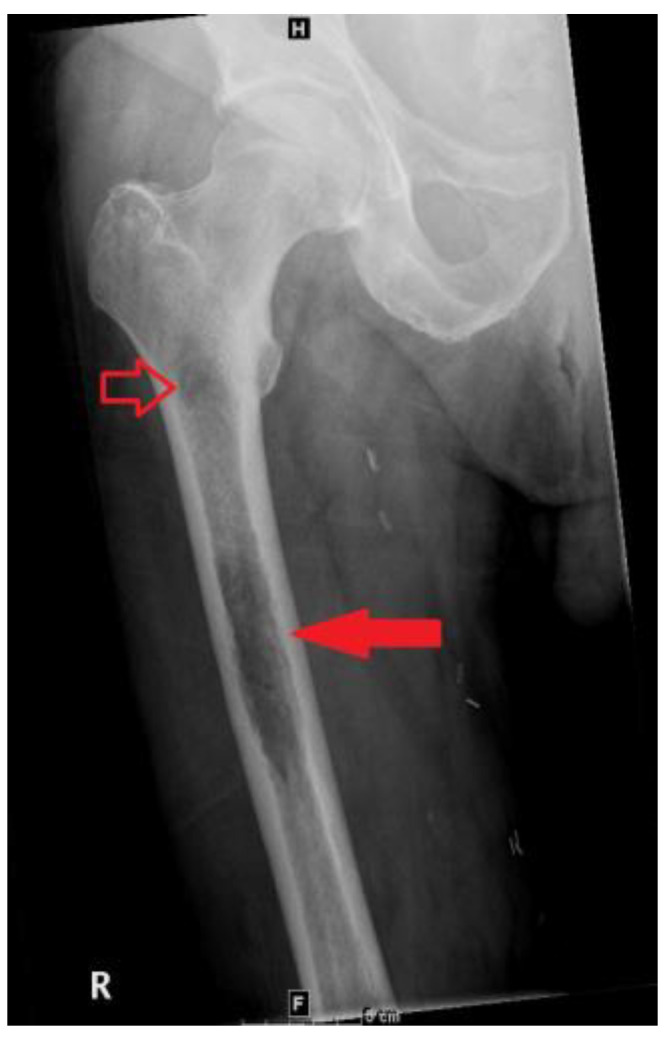
Frontal view radiograph of the right femur demonstrating a 10 cm in length lytic lesion of the proximal half of the femoral diaphysis (solid arrow), with a smaller lesion more proximally (open arrow).

**Figure 3:**
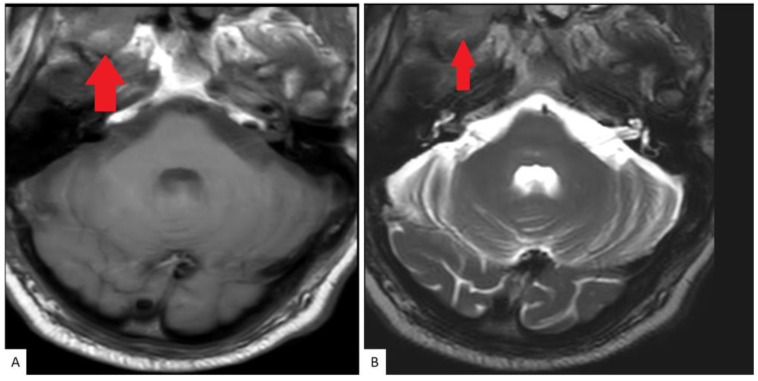
MRI Cervical spine: Axial T1 (**A**) and T2 (**B**) weighted images as part of a cervical spine MRI demonstrate a soft tissue lesion centered in the right maxillary sinus involving the masticator space (red solid arrow).

A bone scan showed a right maxillary sinus mass extending into the orbital floor, as well as increased activity from the proximal right femur to the mid shaft, and the left sacrum. A cardiac MRI revealed an irregularly shaped mass invading the superior vena cava and up to the atrial free wall (Figure 1). The X-ray of the right femur showed a large lytic lesion and multiple small lesions in the proximal right and left femur (Figure 2). Magnetic resonance imaging (MRI) of the brain and cervical spine further confirmed the right maxillary sinus mass extending through the orbital floor and into the pterygopalatine fossa and inferior orbital fissure (Figure 3).

**Figure 4:**
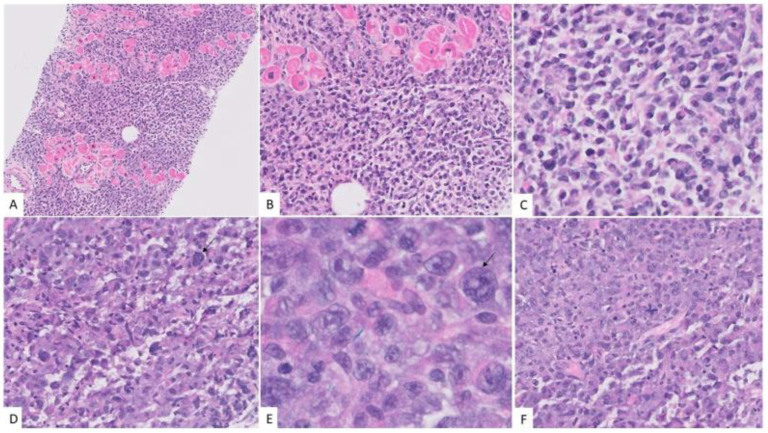
Morphologic features of anaplastic myeloma. The neoplastic cells infiltrated the cardiac muscles. (**A**) Hematoxylin and eosin stain, X40, while some areas had more plasmacytoid cells. (**B**,**C**) Hematoxylin and eosin stain, X100, X400 there were other areas with marked pleomorphism (black arrows) and presence of binucleate cells. (**D**,**E**) Hematoxylin and eosin stain, X400 and 1000X) with basophilic cytoplasm and frequent mitosis. (**F**) Hematoxylin and eosin stain, X400.

A biopsy of the right atrium from endovascular intracardiac transfemoral approach was undertaken in addition to sinus and femoral biopsies. The biopsy revealed sheets of large atypical neoplastic cells, infiltrating the cardiac muscles. While some areas showed sheets of large pleomorphic cells (Figure 4E), some multinucleated and with frequent mitotic figures, there were other areas with more plasmacytoid appearance, with eccentric nucleus and eosinophilic cytoplasm of the cells (Figure 4). 

**Figure 5:**
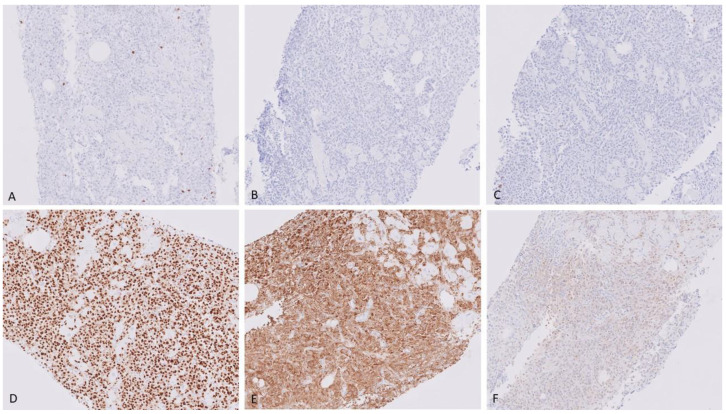
Immunohistochemical stains, showing neoplastic cells negative for CD3 (**A**) X40, CD20 (**B**) X40), and CD138 (**C**) X40. They are positive for MUM1 (**D**) X40, and lambda (**E**) X40. Negative for kappa (**F**) X40.

Neoplastic cells were negative for all immunohistochemical stains in the initial panel which included CD3, CD20, CD138, and AE1/3. Lambda immunoglobulin light chain restriction was positive in the neoplastic cells. An extended panel was performed which showed positivity for CD79a, MUM1, and p53. Cells were negative for CD30, CD43, CD56, CD117, Cyclin D1, HHV8, kappa, and LMP-1 (Figure 5).

**Figure 6:**
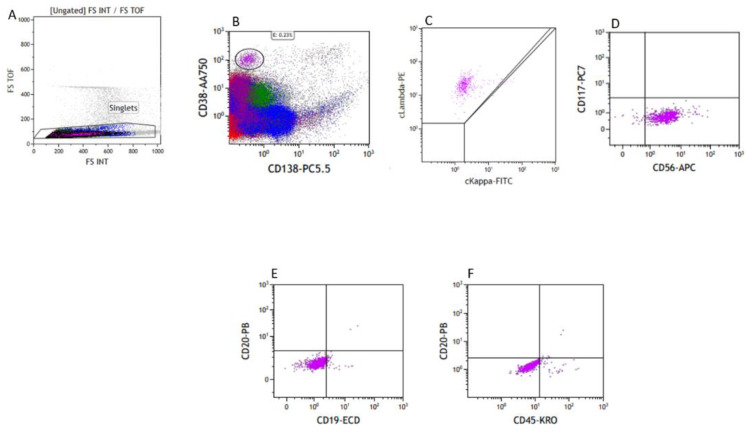
Flow cytometry of the bone marrow, FS-TOF versus FS INT to identify singlets (**A**), with a small population of CD38+/CD138− cells (**B**), Lambda restricted (**C**), CD117−/CD56+ (**D**), CD19−/CD20− (**E**), and dim CD45+ (**F**).

Biopsies from the femur and sinus showed similar plasmacytoid cells with anaplastic morphology. The flow cytometry on the bone marrow showed a very small population of atypical plasma cells, positive for CD38, CD56, and cytoplasmic lambda and negative for CD19, CD20, CD117, CD138, and cytoplasmic Kappa (Figure 6). However, no definite atypical plasma cells were identified on the bone marrow biopsy by MUM1 immunohistochemical stain. 

Most anaplastic myeloma patients have suboptimal response to conventional chemotherapy and radiotherapy. Our patient underwent radiation therapy and chemotherapy with Daratumumab and cyclophosphamide, bortezomib, and dexamethasone (CyBorD). He subsequently underwent an autologous stem cell transplant.

## 3. Discussion 

Multiple myeloma (MM) constitutes 10% of all hematological malignancies [7]. It is characterized by the clonal expansion of malignant plasma cells in the bone marrow. Glycoproteins CD38, CD138, and MUM1 expressed on normal and abnormal plasma cells are used as markers in diagnosing plasma cell neoplasms [5]. CD138 is acquired by B-cells during normal development and is a heparin sulfate proteoglycan that plays a key role in tumor cell proliferation in MM [8]. 

Plasma cell neoplasm with cardiovascular involvement is exceedingly rare. A review of the literature by Guan et al. extending from 1977 to 2020 resulted in 50 cases [9,10,11,12]. Anaplastic myeloma itself is a rare morphological variant of plasma cell myeloma with an aggressive clinical behavior and poor outcome. Though the literature is limited regarding this entity, it is noted to present more often in young patients with extra-medullary disease [3]. Anaplastic myeloma is frequently associated with immunoglobulin A isotype and complex genetic abnormalities identified on karyotyping and fluorescent in situ hybridization (FISH). The common ones include 1q21 amplification, 17p(p53) deletion, t(4;14), and/or chromosome 13 anomalies [13].

Morphologically anaplastic myeloma can present a diagnostic dilemma due to its unusual morphology, especially if it is the initial presentation [14]. This case is particularly unique, since the patient presented with sinus mass and right atrium mass initially instead of the typical C.R.A.B. (hypercalcemia, renal failure, anemia, and bone lesions) features common in most MM patients. Even protein electrophoresis was negative in this case. His raised creatinine was not significant with his history of chronic kidney disease. The imaging study was unusual with involvement of the superior vena cava and the right atrium and another lesion extending from the maxillary sinus to the base of the brain. 

On histopathology, the atrium biopsy showed poorly differentiated anaplastic cells with marked pleomorphism and frequent mitosis. Anaplastic myeloma cells have been described to have marked cytological atypia, pleomorphism, and loss of features which can help identify cell of origin. Therefore, diagnosis is heavily dependent on clinical history, morphology, and immunohistochemical stains [15]. They can mimic high-grade lymphoma or non-hematopoietic malignancy by morphology [15]. The cells often resemble dysplastic megakaryocytes or giant cell osteoclasts [16]. In this case, the initial panel including CD3, CD20, CD138, and AE1/3 were all negative. Luckily, some neoplastic cells revealed plasmacytoid features on close examination, leading to further investigation, and they turned out to be MUM1 positive and monoclonal for lambda immunoglobulin light chain. Flow cytometry of bone marrow biopsy for this patient was initially thought to be negative for monoclonal plasma cells, since only the CD38+/CD138+ plasma cells were analyzed. On further investigation, a small population of CD38+/CD138− lambda restricted atypical cells were noted, further supporting the diagnosis.

The cellular origin of anaplastic myeloma is an immature plasma cell and though they are conventionally CD38+/CD138+, there are cases of CD38-negative and occasional CD138-negative ones, such as in this case reported in the literature [3,4]. CD138 expression level in plasma cell myeloma is variable among cells, but the overall expression is usually high and more consistent than the CD38 marker, therefore cases with CD138 negativity are even more challenging [13]. This emphasizes the fact that if anaplastic myeloma is not considered as a potential diagnosis and limited immunohistochemical markers are used, it can lead to a misdiagnosis. We strongly suggest using CD38, CD138, CD79a, MUM1, and immunoglobulin light chains kappa and lambda, or heavy chains, such as IgG and IgA, if anaplastic myeloma is a possibility. Additionally, studies suggest that low levels of CD138 in MM lead to poorer prognosis and decreased response to treatment with Lenalidomide [17].

Plasmablastic lymphoma is a common differential diagnosis for anaplastic myeloma. It is a rare subtype of B-lymphoid malignancy with pathological features that can overlap with anaplastic myeloma. While clinicopathological features of renal dysfunction, increased paraprotein level, osteolytic lesions, hypercalcemia, and diffuse bone marrow involvement support anaplastic myeloma, EBV positivity and HIV infection favor a diagnosis of plasmablastic lymphoma. Vega et al. in their work found identical immunophenotypic profiles for plasmablastic lymphoma and plasmablastic myeloma, including aberrant plasma cell markers which may be positive in both entities. Therefore, clinical correlation with skeletal involvement of disease and EBV infection are the key features to differentiate the two [3,18].

## 4. Conclusions

This is an unusual presentation of anaplastic myeloma, with multiple lesions involving the heart and base of brain and lacking the typical biochemical findings and immunohistochemistry profile. The diagnosis was based on morphology, lambda light chain restriction, and other plasmocytic markers expression (MUM1 and CD79a). For the unusual staining pattern of the plasma cell (CD38 positive and CD138 negative) additional plasma cell markers, such as MUM1, PRDM1, CD79a, or aberrant markers, such as CD56, CD200, CD28, CD117, CD20, CD52, immunoglobulin light or heavy chains, and CD10, are helpful. Flow-cytometry should be interpreted with caution to identify atypical populations, which includes the CD38+/CD138− population with clonality.

## Data Availability

Data is available in medical records.
